# Stable malaria incidence despite scaling up control strategies in a malaria vaccine-testing site in Mali

**DOI:** 10.1186/1475-2875-13-374

**Published:** 2014-09-19

**Authors:** Drissa Coulibaly, Mark A Travassos, Abdoulaye K Kone, Youssouf Tolo, Matthew B Laurens, Karim Traore, Issa Diarra, Amadou Niangaly, Modibo Daou, Ahmadou Dembele, Mody Sissoko, Bouréima Guindo, Raymond Douyon, Aldiouma Guindo, Bourema Kouriba, Mahamadou S Sissoko, Issaka Sagara, Christopher V Plowe, Ogobara K Doumbo, Mahamadou A Thera

**Affiliations:** Malaria Research & Training Centre, Department of Epidemiology of Parasitic Diseases, Faculty of Medicine and Dentistry, , University of Sciences, Techniques and Technologies, Bamako, Mali; Howard Hughes Medical Institute/Center for Vaccine Development, University of Maryland School of Medicine, Baltimore, MD USA; Centre National de Lutte contre la drépanocytose, Ministère de la Santé, Bamako, Mali; Laboratoire de Parasitologie et mycologie médicale, Université Claude Bernard Lyon 1, Villeurbanne, France

**Keywords:** Malaria incidence, Malaria parasite prevalence, *Plasmodium falciparum*

## Abstract

**Background:**

The recent decline in malaria incidence in many African countries has been attributed to the provision of prompt and effective anti-malarial treatment using artemisinin-based combination therapy (ACT) and to the widespread distribution of long-lasting, insecticide-treated bed nets (LLINs). At a malaria vaccine-testing site in Bandiagara, Mali, ACT was introduced in 2004, and LLINs have been distributed free of charge since 2007 to infants after they complete the Expanded Programme of Immunization (EPI) schedule and to pregnant women receiving antenatal care. These strategies may have an impact on malaria incidence.

**Methods:**

To document malaria incidence, a cohort of 400 children aged 0 to 14 years was followed for three to four years up to July 2013. Monthly cross-sectional surveys were done to measure the prevalence of malaria infection and anaemia. Clinical disease was measured both actively and passively through continuous availability of primary medical care. Measured outcomes included asymptomatic *Plasmodium* infection, anaemia and clinical malaria episodes.

**Results:**

The incidence rate of clinical malaria varied significantly from June 2009 to July 2013 without a clear downward trend. A sharp seasonality in malaria illness incidence was observed with higher clinical malaria incidence rates during the rainy season. Parasite and anaemia point prevalence also showed seasonal variation with much higher prevalence rates during rainy seasons compared to dry seasons.

**Conclusions:**

Despite the scaling up of malaria prevention and treatment, including the widespread use of bed nets, better diagnosis and wider availability of ACT, malaria incidence did not decrease in Bandiagara during the study period.

## Background

In the past few decades, new tools and strategies for malaria control such as artemisinin-based combination therapy (ACT), long-lasting insecticide treated bed nets (LLINs), intermittent preventive treatment in pregnancy (IPTp), and intermittent preventive treatment in infancy (IPTi) and seasonal malaria chemoprevention (SMC) in children showed efficacy in malaria control [1, 2]. Declines in the malaria burden in many areas have been attributed largely to the scaling up of these interventions [3].

In Mali, ACT was introduced in 2004 and LLINs have been distributed free of charge since 2007 to infants aged less than one year upon completion of their routine Expanded Programme of Immunization (EPI) schedule. These interventions have been routinely available in the Malian town of Bandiagara since 2007. This town has been the testing site for several malaria vaccine candidates since 2003 [4–7]. The impact of studies on the reduction of the malaria burden at other clinical trial sites has been reported [8–10]. The clinical trials conducted in Bandiagara have been planned on the basis of malaria incidence data collected in 1999 to estimate sample sizes [11]. Updated information on malaria infection and clinical disease in the context of scaled-up malaria control interventions is needed to plan new clinical trials of malaria vaccines and other interventions and to estimate sample sizes at this site.

The main objective of this study was to measure *Plasmodium falciparum* malaria clinical disease in children over the course of several consecutive malaria seasons (from 2009 to 2013) in Bandiagara, Mali. The seasonal prevalence of malaria infection and anaemia in children and infants was also determined over this study period.

## Methods

### Study area

The study was conducted in Bandiagara, a town of approximately 14,000 inhabitants located in central Mali, on a rocky plain. The mean annual rainfall is about 600 mm. A small river, the Yame, runs through Bandiagara town. This river is a minor tributary of the Niger that flows during the rainy season, and holds transient post-rainfall standing water the rest of the year. *Anopheles gambiae s.l.* complex are the main malaria vectors. Malaria transmission is highly seasonal, with minimal transmission during the dry season in March with essentially no detectable infective mosquito bites. Transmission occurs during the rainy season from July to November with a peak of up to 60 infective mosquito bites per person per month in August or September [12, 13]. *Plasmodium falciparum* represents 97% of malaria infections, with the remaining 3% due mostly to *Plasmodium malariae* and rare *Plasmodium ovale* infections.

### Subject recruitment and enrolment

After obtaining community permission as described by Diallo *et al.* [14], the study was publicized by local radio broadcast. Two rounds of participant recruitment were done. Parents were invited to accompany children to the Bandiagara Malaria Project (BMP) Research Clinic to be screened for eligibility. Participants were enrolled on a first-come, first-served basis until the target number in each age strata was reached. In June 2009, a subcohort of 300 children under six years of age was enrolled. Another subcohort of 100 children between seven and 14 years of age was enrolled in June 2010. One-hundred children were included in each of four age categories: under two years, three to four years, five to six years, and seven to 14 years to permit age-specific malaria incidence rate measurement.

### Inclusion and exclusion criteria

Children in the target age group were eligible for inclusion in the study if they met all of the following criteria: residency in Bandiagara town, general good health based on clinical evaluation, aged under 14 years inclusive at the time of screening, written informed consent obtained from the parent/guardian, assent from children aged 13 years and above, and availability to participate in follow-up for the duration of study. Exclusion criteria were: simultaneous participation in an interventional clinical trial and chronic use of a medication with known anti-malarial activity, such as trimethoprim-sulphamethoxazole.

### Ethical clearance

The study protocol and informed consent/assent process were reviewed and approved by the institutional review boards of the Faculty of Medicine, Pharmacy and Dentistry of the University of Sciences, Techniques and Technologies of Bamako and the University of Maryland School of Medicine. Permission to work in the community was obtained from local official authorities. Individual written informed consent was obtained from parents or guardians and assent was obtained from children aged 13 years and older.

### Study procedures

Active surveillance consisted of scheduled monthly visits (every four weeks) with the aim of detecting asymptomatic malaria infection and anaemia. At each visit, participants were questioned for symptoms of malaria and examined. Finger-prick blood was collected for malaria thick smear and haemoglobin level. Smears were not read contemporaneously unless symptoms or signs of malaria were present. At enrolment, glucose-6-phoshate dehydrogenase (G6PD) deficiency and haemoglobin type were determined. Passive surveillance consisted of continuous availability of free, basic medical care at the BMP Research Clinic and Bandiagara District Hospital. Parents/guardians were encouraged to visit the clinic at any time if their child became sick. Children were examined by a physician. If symptoms or signs compatible with malaria were present, finger-prick blood was collected for a blood film, which was read immediately. Uncomplicated malaria was treated with ACT (artesunate/amodiaquine or artemether/lumefantrine) according to the guidelines of the Mali National Malaria Control Programme. Parents/guardians were instructed to administer only drugs given or prescribed by the research team. Two definitions for clinical malaria were used: (i) a broader definition based on treatment-seeking behaviour, clinical symptoms consistent with malaria and presence of malaria parasites at any density; and, (ii) a more specific definition with parasite density ≥2,500 parasites per microlitre, body temperature ≥37.5°C, and symptoms consistent with malaria such as fever, headaches, joint pain, vomiting, diarrhoea, or abdominal pain. Anaemia was defined as a haemoglobin level <10 g/dL.

### Sample size estimation

To adequately estimate malaria incidence, an average of 0.75 clinical malaria episodes per subject per year, a confidence level of 95%, and a Poisson distribution of malaria were assumed. With the above assumption, 400 total subjects, 100 subjects in each age category (under two years, three to four years, five to six years, and seven to 14 years) were needed to estimate the age category-specific malaria incidence with a confidence interval of +/− 0.20 clinical episodes per subject per year.

### Laboratory assays

Malaria thick smears were Giemsa-stained and parasites counted against 300 leukocytes to give parasite counts/mm^3^, assuming a leukocyte count of 7,500/mm^3^. Standard operating procedures were developed to ensure uniform and high-quality malaria smears, including training and qualifying malaria microscopists. Haemoglobin type was determined by high-performance liquid chromatography (D-10 instrument; Bio-Rad). Restriction fragment length polymorphism analysis was used to identify the (A-) allele of the G6PD deficiency, as previously described (15). Haemoglobin determinations were made using Hemocue haemoglobin analyzers (Hemocue Inc, Cypress, CA, USA).

### Statistical methods

Data were double-entered and then reconciled using Microsoft ACCESS 2007. The analysis was performed using STATA software, version 12 (Stata Corp, College Station, TX, USA). Descriptive statistics were used to summarize baseline values and demographic characteristics (age, gender, ethnicity, and neighbourhood). Pearson Chi-square tests or exact probabilities statistics were used to compare categorical variables. Multivariate analysis using Poisson regression was performed to estimate the risk ratio of malaria episode adjusting for covariates. All p values <0.05 were considered statistically significant. Age-specific incidence rates (the number of episodes per person over the study period) of clinical malaria were calculated. Clinical malaria episodes separated by at least 14 days were considered distinct, separate illnesses. The follow-up time was four consecutive years for participants under six years old and three consecutive years for participants aged seven to 14 years.

## Results

### Sociodemographic and baseline characteristics

A total of 519 participants were screened (405 under six years old and 114 aged seven to 14 years). Of these, 400 that met inclusion criteria were enrolled, (300 aged under six years and 100 aged seven to 14 years). Main reasons for exclusion were willingness to travel out of the study area and parents’ refusal. The sex ratio was 0.9 (191 boys *vs* 209 girls, (Table 1)). The mean age was 3.6 years (95% CI (3.4-3.8)) for children aged under six years and 11 years (95% CI (10.58-11.42)) for older children. Dogon was the main ethnic group (74%). Haemoglobin AA was the most frequent phenotype (78.8%). Other phenotypes include haemoglobin AC (13%), haemoglobin AS (6.3%), haemoglobin CC (1.2%), and haemoglobin SC (0.7%) (Table 2). Thirty-eight participants (9.5%) had G6PD deficiency (Table 3). Among G6PD-deficiency carriers, 17% were hemizygous, 16% heterozygous and 5% homozygous.

**Table 1:** **Age category and gender distribution**

Sex	0-2 years	3-4 years	5-6 years	7-10 years	>10 years	Total
Male	49	40	53	22	27	191
Female	39	58	61	14	37	209
Total	88	98	114	36	64	400

**Table 2:** **Haemoglobin type distribution per ethnic group**

Ethnics	AA	AC	AS	CC	SC	Total
Bambara	15	3	1	0	1	20
Dogon	230	44	15	5	1	295
Peulh	31	1	4	0	0	36
Sonrhaï	15	2	3	0	1	21
Others	24	2	2	0	0	28
Total	315 (78.8%)	52 (13%)	25 (6.3%)	5 (1.2%)	3 (0.7%)	400

**Table 3:** **G6PD deficiency distribution per ethnic group**

Ethnics	Normal	Deficiency	Total (%)
Bambara	20	0	20
Dogon	266	29	295
Peulh	32	4	36
Sonrhaï	19	2	21
Others	25	3	28
Total	362 (90.5%)	38 (9.5%)	400

### Clinical malaria

With malaria defined using a minimum parasite density threshold, 1,193 episodes of clinical malaria were encountered. When a broader malaria case definition was used irrespective of body temperature at the time of clinic visit, a total of 1,908 clinical episodes were documented (1,494 and 414 clinical malaria episodes in the younger and older subcohorts, respectively). Among children aged under six years, 85.7% of those who experienced clinical malaria had fever. In patients diagnosed with clinical malaria, symptoms included history of fever (90.7%), headache (65.5%), vomiting (34.3%), abdominal pain (17.7%), and diarrhoea (6.7%). Approximately 97% (1,842/1,908) of children reported sleeping under a bed net since their previous scheduled visit. Most of these bed nets (90.9%) were insecticide-treated.

Overall, six severe malaria cases occurred during the study, all in children aged three to 11 years. Their symptoms were coma in two cases, seizures in three cases and lethargy in one case. All had normal haemoglobin (AA) and normal G6PD status.

**Figure 1:**
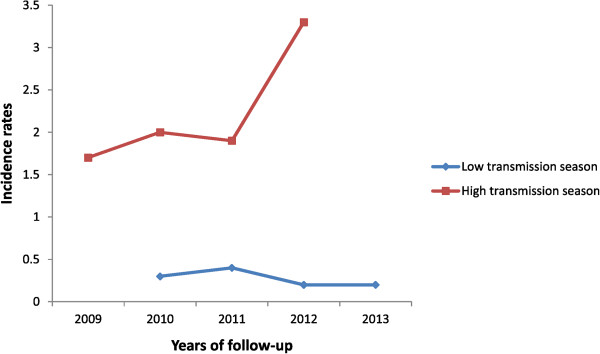
**Malaria Incidence rate by year and per transmission season.**

The overall annual incidence rate was 1.4 clinical malaria episodes (clinical symptoms consistent with malaria and presence of parasite at any density) during study period. The incidence was much higher from June to December compared to the levels observed from January to May (Table 4). The annual incidence rates were 1.7, 2.0, 1.9, and 3.3, respectively, in transmission seasons 2009, 2010, 2011, and 2012 (Figure 1). There was an association between malaria incidence and age in 2009. The youngest children (aged less than two years old) had fewer episodes of clinical malaria compared to those aged at least five years (RR = 0.63, 95% CI (0.46-0.85); *p = 0.002*). No similar association between malaria incidence and age was observed in years that followed. Also, haemoglobin type C was associated with protection against malaria (RR = 0.59, 95% CI (0.40-0.89); *p = 0.012*). G6PD deficiency was not associated with protection against clinical malaria (RR = 0.88, 95% CI (0.60-1.30); *p = 0.539*) (Table 5).

**Table 4:** **Malaria Incidence rate by year and per transmission season**

Years	Transmission season**	Number of cases	Person-year	Incidence rate	*IRR (95% CI)	p
2009***	High transmission season	276	157	1.7		Ref
2010	Overall	456	324	1.4		
	Low transmission season	32	115	0.3		
	High transmission season	424	208	2	1.15 (0.99–1.35)	0.05
2011	Overall	458	352	1.3		
	Low transmission season	58	149	0.4		
	High transmission season	400	202	1.9	1.12 (0.96–1.31)	0.11
2012	Overall	672	335	2		
	Low transmission season	25	141	0.2		
	High transmission season	647	196	3.3	1.87 (1.62–2.16)	<0.001
2013	Low transmission season	46	192	0.2		

*IRR = Incidence rate ratio for high transmission season; 95% CI is 95% confidence interval.

**Transmission season; High transmission season was from June-December (rainy season) and low transmission season was from January-May (dry season).

***2009 high transmission season was used as reference (Ref) in estimating IRR for other years during high transmission season.

**Table 5:** **Incidence rate ratio estimates in year 2009 adjusting for covariates**

Covariates	IRR* (95% CI)	p
**3-4 years**	0.93 (0.70-1.23)	0.623
**<2 years**	0.63 (0.46-0.85)	0.002
**G6PD deficiency**	0.88 (0.60-1.30)	0.539
**Haemoglobin C**	0.59 (0.40-0.89)	0.012
**Haemoglobin AS**	0.78 (0.42-1.45)	0.442

*IRR: Incidence rate ratio estimated using Poisson regression; ≥5 years old, normal G6PD deficiency and normal haemoglobin type were used as reference. CI is the 95% confidence interval.

### Prevalence of infection

**Figure 2:**
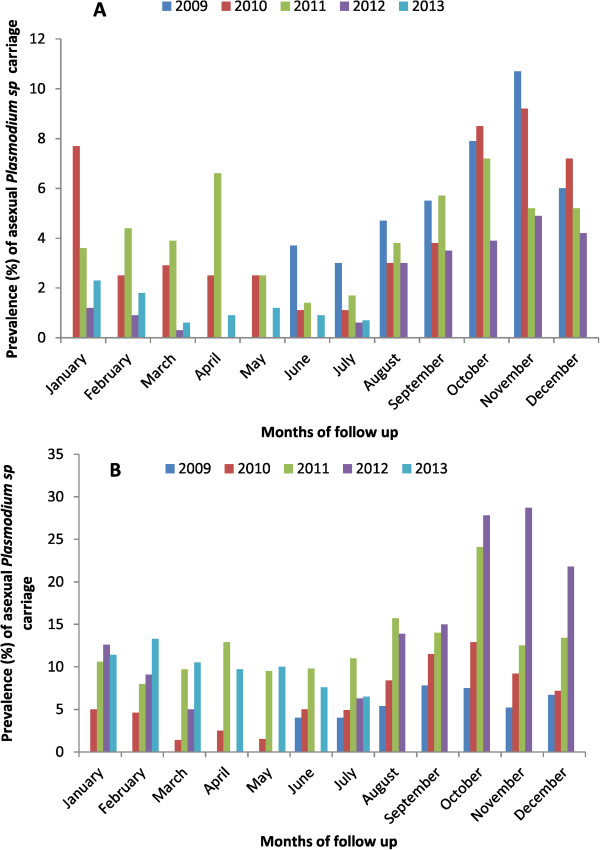
**Monthly prevalence of asexual** ***Plasmodium sp.*** **carriage per year and per age categories A (children under 5 years old) B (children above 5 years old).**

The highest average point prevalence of parasitaemia were observed from August to November of each study year for all age categories. The lowest prevalence of infection observed was 3, 1.1, 1.4, 0.3 and 0.6%, respectively, in 2009, 2010, 2011, 2012, and 2013. The highest prevalence of infection was 10.7, 12.9, 24.1, 28.7, and 13.3%, respectively, in 2009, 2010, 2011, 2012, and 2013 (Figure 2).

### Prevalence of anaemia

**Figure 3:**
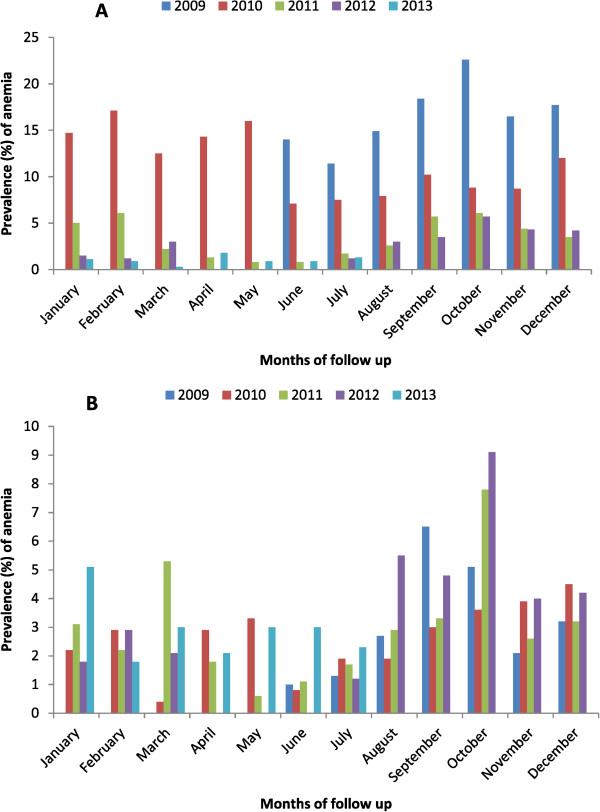
**Monthly prevalence of anaemia per year and per age categories A (children under 5 years old) B (children above 5 years old).**

Monthly time point prevalence of anaemia was 19.7, 13.8, 6.2, 6.9, and 24.9%, respectively, in 2009, 2010, 2011, 2012, and 2013 (Figure 3). The highest prevalence of anaemia was observed from September to October for all study years. For instance, in October 2009 the younger children (<five years old) were more affected compared to the older age group (≥five years old); the anaemia prevalence was 22.6% (66/292) and 5.1% (15/292), respectively, *p < 0.001*.

## Discussion

Decreases in malaria incidence have been observed in parts of Africa following prompt and effective anti-malarial treatment with ACT in conjunction with the widespread use of LLINs [1, 2]. Bandiagara, Mali has been developed as a site for testing malaria vaccines. In the context of nationwide scale up of malaria control efforts, it was necessary to update age-specific incidence of malaria infection for sample size determination of future efficacy trials of malaria vaccine candidates and other interventions at this site.

A closed cohort of 400 children was followed using active and passive surveillance over four years. Continuous availability of free, expeditious, high-quality medical care, including rapid microscopic diagnosis of malaria, along with study investigators long experience in the site and good rapport with the population, provided confidence that the follow-up procedures captured all clinical malaria episodes that occurred in the cohort during the study period. For the purpose of analyses, two age strata were defined, with an age threshold set at five years. This allowed to consider the group of children aged less than five years, which represent the most vulnerable group to malaria. Children under five years old are also the target population for World Health Organization policy recommendation on the use of SMC [15].

Annual clinical malaria incidence was remarkably stable in this well-characterized and closely followed cohort over four years. Moreover, a statistically significant increase in clinical malaria incidence was reported in the 2012 transmission season, probably due to more abundant rainfall that year. These results contrast with data recently published that showed a significant decrease in malaria burden in Senegal [10], Kenya [16], Rwanda [17], and Zambia [18]. A systematic review covering data from 39 studies conducted in 16 African countries from 1987 to 2007 compared the proportion of fever associated with *P. falciparum* before 2000 and afterwards. The proportion of fever associated with *P. falciparum* decreased by 50% in the period after 2000 [19]. However, a closer look at the data from Kenya showed a more complex pattern, with districts where malaria burden was reduced and others where malaria-associated hospital admissions sharply increased. In addition, changes in malaria burden appear to have occurred before scaling up of malaria control interventions [20]. There have been increases in malaria-associated hospital admissions in Uganda [21]. Thus decreases in the malaria burden following scaling up of malaria interventions in Africa have not occurred uniformly. The factors affecting measures of malaria burden may be complex - for example, initial increases in hospital admissions reported in some countries could in part reflect changing treatment-seeking behaviour patterns due to health education and outreach that ultimately lead to substantial decreases in disease burden as it has been described in Senegal [10].

Data from this cohort differ from large population-based studies and several hospital record-based analyses used in others studies. If the town of Bandiagara achieved high coverage of control interventions, this should have produced an impact that would also be observed in this cohort. Achieving universal coverage in control interventions has been advocated by Roll Back Malaria since 2005 [22, 23]. The level of coverage in malaria control interventions in Bandiagara, a town of 14,000 inhabitants has not been systematically determined for this study. The Malian National Malaria Control Programme started implementing its new strategy in 2007. This new strategy included free nationwide distribution of LLINs to all children aged less than five years and to all pregnant women, malaria treatment based on ACT and rapid diagnostic tests free of charge for all children aged less than five years. In its 2012 report, the Mali National Malaria Control Programme documented 96% coverage of the target population in Bandiagara with the primary control interventions (LLINs and IPTp) [24]. Stable incidence of malaria reported here was determined in a cohort that benefited from the same interventions that were carried out in the larger community in Bandiagara. In addition, the researcher verified that all children from the cohort benefited from the National Malaria Control Programme interventions to avoid any ethical injustice because of their inclusion in the study.

It is noteworthy that during the 2000–2010 decade, the town of Bandiagara benefited from major urbanization with new infrastructure, such as new dwellings, asphalt-paved roads, rainwater drain canals, electricity, and water supply. These changes have profoundly affected the local micro-epidemiology of malaria. Clinical malaria cases are distributed in time-space clusters in Bandiagara [25]. The characteristics of these clusters are determined by the local conditions created by the environment change at a local scale; these clusters may be prone to malaria transmission or instead may be unfavourable to it depending on the effects of these infrastructure changes.

Bandiagara has a Sahelian setting with annual rainfall concentrated over three months and a long dry season where previous studies have detected no infected mosquitoes. Malaria transmission is sharply seasonal. In this cohort, follow-up was continued during the dry season, and a persistence of clinical malaria that was not reported before, was observed. The incidence rate was much lower, with approximately one clinical episode of malaria occurring once in every five children, but at this low level, malaria transmission persisted throughout the dry season.

Classically, malaria incidence is higher in younger children in high transmission settings [26]. In this study, less clinical malaria was seen in younger children as compared to those aged more than five years during the first year of follow-up only. This observation is supported by other data, that as malaria transmission decreases, there is a shift in the age group that bears the highest disease burden [27]. Other entomological parameters of transmission were not concomitantly measured. Parasite prevalence rates regularly assessed are lower than levels determined several years ago at the same site [11]. WHO recommends that SMC be applied to children aged under five years old in areas such as Bandiagara, where 60% of malaria transmission is concentrated in three to four months each year. The results reported here call for an extension of the SMC age group to include children older than five years old. Others have reached similar conclusions using modelling approaches on large-scale data sets [26]. The upper level of the age group was not determined in this study. Children up to 15 years old were enrolled. However, it is likely that the adult population in Sahelian regions bear a heavy malaria burden [28]. More data on malaria distribution in this older age group are urgently needed in African Sahelian countries in view of increased efforts at malaria elimination.

## Conclusion

These results indicate an absence of decline in malaria incidence following the scaling up of malaria prevention and control measures since 2004, including the widespread use of LLINs, better diagnostics and a wider availability of ACT. Malaria transmission was seasonal in Bandiagara; 93% of cases occurred from June to December. In view of the prospect of large-scale implementation of SMC, a new WHO-recommended control strategy for Sahelian countries is important to re-examine the target age group and consider extending it to include children older than five years old.

## References

[1] Greenwood BM, Fidock DA, Kyle DE, Kappe SH, Alonso PL, Collins FH, Duffy PE (2008). Malaria: progress, perils, and prospects for eradication. J Clin Invest.

[2] WHO (2011). World Malaria Report 2011.

[3] WHO (2013). World Malaria Report 2013.

[4] Thera MA, Doumbo OK, Coulibaly D, Diallo DA, Sagara I, Dicko A, Diemert DJ, Heppner DG, Stewart VA, Angov E, Soisson L, Leach A, Tucker K, Lyke KE, Plowe CV, for the Mali FMP1 Working Group (2006). Safety and allele-specific immunogenicity of a malaria vaccine in Malian adults: results of a phase I randomized trial. PLoS Clin Trials.

[5] Thera MA, Doumbo OK, Coulibaly D, Diallo DA, Sagara I, Dicko A, Diemert DJ, Heppner DG, Stewart VA, Angov E, Soisson L, Leach A, Tucker K, Lyke KE, Plowe CV, for the Mali FMP1 Working Group (2008). Safety and immunogenicity of an AMA-1 malaria vaccine in Malian adults: results of a phase 1 randomized controlled trial. PLoS One.

[6] Thera MA, Doumbo OK, Coulibaly D, Laurens MB, Kone AK, Guindo AB, Traore K, Sissoko M, Diallo DA, Diarra I, Kouriba B, Daou M, Dolo A, Baby M, Sissoko MS, Sagara I, Niangaly A, Traore I, Olotu A, Godeaux O, Leach A, Dubois MC, Ballou WR, Cohen J, Thompson D, Dube T, Soisson L, Diggs CL, Takala SL, Lyke KE (2010). Safety and immunogenicity of an AMA1 malaria vaccine in Malian children: results of a phase 1 randomized controlled trial. PLoS One.

[7] Thera MA, Doumbo OK, Coulibaly D, Laurens MB, Ouattara A, Kone AK, Guindo AB, Traore K, Traore I, Kouriba B, Diallo DA, Diarra I, Daou M, Dolo A, Tolo Y, Sissoko MS, Niangaly A, Sissoko M, Takala-Harrison S, Lyke KE, Wu Y, Blackwelder WC, Godeaux O, Vekemans J, Dubois MC, Ballou WR, Cohen J, Thompson D, Dube T, Soisson L (2011). A field trial to assess a blood-stage malaria vaccine. N Engl J Med.

[8] O'Meara WP, Bejon P, Mwangi TW, Okiro EA, Peshu N, Snow RW, Newton CR, Marsh K (2008). Effect of a fall in malaria transmission on morbidity and mortality in Kilifi, Kenya. Lancet.

[9] O'Meara WP, Mangeni JN, Steketee R, Greenwood B (2010). Changes in the burden of malaria in sub-Saharan Africa. Lancet Infect Dis.

[10] Trape JF, Tall A, Sokhna C, Ly AB, Diagne N, Ndiath O, Mazenot C, Richard V, Badiane A, Dieye-Ba F, Faye J, Ndiaye G, Diene Sarr F, Roucher C, Bouganali C, Bassène H, Touré-Baldé A, Roussilhon C, Perraut R, Spiegel A, Sarthou JL, da Silva LP, Mercereau-Puijalon O, Druilhe P, Rogier C (2014). The rise and fall of malaria in a West African rural community, Dielmo, Senegal, from 1990 to 2012: a 22 year longitudinal study. Lancet Infect Dis.

[11] Coulibaly D, Diallo DA, Thera MA, Dicko A, Guindo AB, Koné AK, Cissoko Y, Coulibaly S, Djimdé A, Lyke K, Doumbo OK, Plowe CV (2002). Impact of preseason treatment on incidence of falciparum malaria and parasite density at a site for testing malaria vaccines in Bandiagara, Mali. Am J Trop Med Hyg.

[12] Lyke KE, Dicko A, Kone A, Coulibaly D, Guindo A, Cissoko Y, Traoré K, Plowe CV, Doumbo OK (2004). Incidence of severe *Plasmodium falciparum* malaria as a primary endpoint for vaccine efficacy trials in Bandiagara, Mali. Vaccine.

[13] Takala SL, Coulibaly D, Thera MA, Dicko A, Smith DL, Guindo AB, Kone AK, Traore K, Ouattara A, Djimde AA, Sehdev PS, Lyke KE, Diallo DA, Doumbo OK, Plowe CV (2007). Dynamics of polymorphism in a malaria vaccine antigen at a vaccine-testing site in Mali. PLoS Med.

[14] Diallo DA, Doumbo OK, Plowe CV, Wellems TE, Emanuel EJ, Hurst SA (2005). Community permission for medical research in developing countries. Clin Infect Dis.

[15] WHO/Global Malaria Programme (2012). WHO policy recommendation. Seasonal Malaria Chemoprevention (SMC) for Plasmodium Falciparum Malaria Control in Highly Seasonal Transmission Areas of the Sahel Sub-Region in Africa.

[16] Okiro EA, Hay SI, Gikandi PW, Sharif SK, Noor AM, Peshu N, Marsh K, Snow RW (2007). The decline in paediatric malaria admissions on the coast of Kenya. Malar J.

[17] Karema C, Aregawi MW, Rukundo A, Kabayiza A, Mulindahabi M, Fall IS, Gausi K, Williams RO, Lynch M, Cibulskis R, Fidele N, Nyemazi JP, Ngamije D, Umulisa I, Newman R, Binagwaho A (2012). Trends in malaria cases, hospital admissions and deaths following scale-up of anti-malarial interventions, 2000–2010. Rwanda Malar J.

[18] Masaninga F, Chanda E, Chanda-Kapata P, Hamainza B, Masendu HT, Kamuliwo M, Kapelwa W, Chimumbwa J, Govere J, Otten M, Fall IS, Babaniyi O, Siziya S (2013). Review of the malaria epidemiology and trends in Zambia. Asian Pac J Trop Biomed.

[19] D'Acremont V, Lengeler C, Genton B (2010). Reduction in the proportion of fevers associated with *Plasmodium falciparum* parasitaemia in Africa: a systematic review. Malar J.

[20] Okiro EA, Alegana VA, Noor AM, Snow RW (2010). Changing malaria intervention coverage, transmission and hospitalization in Kenya. Malar J.

[21] Okiro EA, Bitira D, Mbabazi G, Mpimbaza A, Alegana VA, Talisuna AO, Snow RW (2011). Increasing malaria hospital admissions in Uganda between 1999 and 2009. BMC Med.

[22] Coll-Seck AM (2008). A golden age for malaria research and innovation. Malar J.

[23] Nafo TF (2005). Rolling back malaria: opportunities and challenges. Trans R Soc Trop Med Hyg.

[24] Mali PNLP (2012). Rapport Annuel 2012.

[25] Coulibaly D, Rebaudet S, Travassos M, Tolo Y, Laurens M, Kone AK, Traore K, Guindo A, Diarra I, Niangaly A, Daou M, Dembele A, Sissoko M, Kouriba B, Dessay N, Gaudart J, Piarroux R, Thera MA, Plowe CV, Doumbo OK (2013). Spatio-temporal analysis of malaria within a transmission season in Bandiagara. Mali Malar J.

[26] Griffin JT, Ferguson NM, Ghani AC (2014). Estimates of the changing age-burden of *Plasmodium falciparum* malaria disease in sub-Saharan Africa. Nat Commun.

[27] Carneiro I, Roca-Feltrer A, Griffin JT, Smith L, Tanner M, Schellenberg JA, Greenwood B, Schellenberg D (2010). Age-patterns of malaria vary with severity, transmission intensity and seasonality in sub-Saharan Africa: a systematic review and pooled analysis. PLoS One.

[28] Guillebaud J, Mahamadou A, Zamanka H, Katzelma M, Arzika I, Ibrahim ML, Eltahir EA, Labbo R, Druilhe P, Duchemin JB, Fandeur T (2013). Epidemiology of malaria in an area of seasonal transmission in Niger and implications for the design of a seasonal malaria chemoprevention strategy. Malar J.

